# FIB Secondary Etching Method for Fabrication of Fine CNT Forest Metamaterials

**DOI:** 10.1007/s40820-017-0145-5

**Published:** 2017-04-08

**Authors:** Adam Pander, Akimitsu Hatta, Hiroshi Furuta

**Affiliations:** 1 Department of Electronic and Photonic Systems Engineering, Kochi University of Technology, Tosayamada, Kami, Kochi, 782-8502 Japan; 2 Center for Nanotechnology, Research Institute, Kochi University of Technology, Tosayamada, Kami, Kochi, 782-8502 Japan

**Keywords:** Carbon nanotubes, Metamaterial, FIB patterning, Secondary etching method, Chemical vapor deposition

## Abstract

Anisotropic materials, like carbon nanotubes (CNTs), are the perfect substitutes to overcome the limitations of conventional metamaterials; however, the successful fabrication of CNT forest metamaterial structures is still very challenging. In this study, a new method utilizing a focused ion beam (FIB) with additional secondary etching is presented, which can obtain uniform and fine patterning of CNT forest nanostructures for metamaterials and ranging in sizes from hundreds of nanometers to several micrometers. The influence of the FIB processing parameters on the morphology of the catalyst surface and the growth of the CNT forest was investigated, including the removal of redeposited material, decreasing the average surface roughness (from 0.45 to 0.15 nm), and a decrease in the thickness of the Fe catalyst. The results showed that the combination of FIB patterning and secondary etching enabled the growth of highly aligned, high-density CNT forest metamaterials. The improvement in the quality of single-walled CNTs (SWNTs), defined by the very high G/D peak ratio intensity of 10.47, demonstrated successful fine patterning of CNT forest for the first time. With a FIB patterning depth of 10 nm and a secondary etching of 0.5 nm, a minimum size of 150 nm of CNT forest metamaterials was achieved. The development of the FIB secondary etching method enabled for the first time, the fabrication of SWNT forest metamaterials for the optical and infrared regime, for future applications, e.g., in superlenses, antennas, or thermal metamaterials.

**Electronic supplementary material:**

The online version of this article (doi:10.1007/s40820-017-0145-5) contains supplementary material, which is available to authorized users.

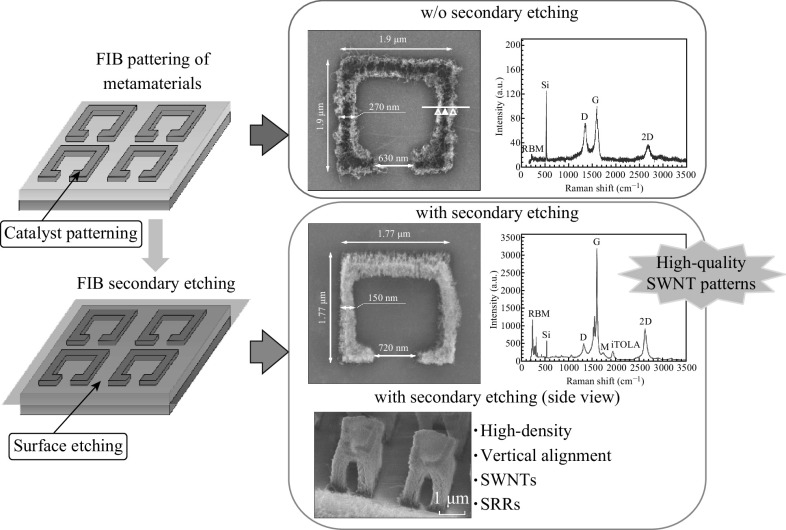

## Highlights

- A newly developed FIB secondary etching method enables the precise fabrication of SWNT forest patterns in sizes from hundreds of nanometers to several micrometers, for the first time. This method fills the previous fabrication gap in this range.
- The precise control of the CNT forest structure is achieved by controlling the FIB processing parameters.
- By applying FIB secondary etching, the growth of the FIB-patterned CNT forest is significantly improved, enabling them to be used in metamaterial, electronic, photonic, and thermal applications.

## Introduction

Carbon nanotubes (CNTs) possess extraordinary electrical, physical and optical properties [1–5] due to their unique structure, such as the anisotropic electrical conductivity of horizontally aligned CNTs [2] and the anisotropic optical absorption of vertically aligned CNTs [3]. The extraordinary properties of CNT forests were recently used for the fabrication of metamaterials and nonlinear photonic devices in the form of multi-walled CNT (MWNT) arrays [6–8], slits cut in the CNT films [9], or spray-coated CNT films on ceramic metamaterials [10, 11]; however, none of these examples utilized the unique properties of a CNT forest. To fully utilize the properties of a CNT forest with the high alignment, density, and the CNT forest structure, a different method of patterning is required. Patterned growth of CNTs on the scale of tenths of micrometers has been successfully reported on silica [12–14], silicon substrates [15], and also on predeposited catalyst [16–18]. On the other hand, the patterning of predeposited catalyst at the nanoscale, which would enable uniform, vertically aligned SWNT forest growth on various shapes of nanosize structures, is still challenging and is yet to be achieved [17, 19–21]. High-quality CNT structures with various shapes were achieved by utilizing different fabrication methods. Standard lithography methods, like photolithography [16, 22], electron beam lithography (EBL) [23], or soft-lithography [24] enabled the growth of fine CNT patterns. A laser machining method was used for the fabrication of one-dimensional grating patterns [25], while a laser etching method was utilized to obtain microstructures of CNT brushes [21]. Moreover, the patterned growth of CNTs was also achieved using anodic aluminum oxide (AAO) templates [26] and inkjet printing of the catalyst [18]. Finally, a focused ion beam (FIB) and electron beam (EB) were used for the patterning of catalyst nanodots and enabled the fabrication of arrays of individual MWNTs [6, 8, 27].

For the patterning of nanostructures on the predeposited catalyst, high precision at the nanoscale is required. The ability to pattern thin films by the FIB method has been presented before and has been successfully used to fabricate metamaterial nanostructures from metals [28]. The FIB method is maskless and allows the fabrication of nanoscale patterns of various shapes and sizes; however, due to sputtering, the redeposited material is observed on the surface outside the patterned area [29]. In this study, in order to overcome the major disadvantage of the redeposition of material, a FIB secondary etching method was developed to clean the patterned surface from the redeposited material by low-depth ion beam irradiation [29] and to improve the growth of CNTs in patterned areas.

The purpose of this study is to provide a reliable method for the precise fabrication of CNT patterns for metamaterials, with sizes ranging from around 150 nm to single micrometers, for the optical and infrared regime, and future applications, e.g., in superlenses, antennas, and thermal metamaterials. In this work, the influence of FIB fabrication parameters on the catalyst surface morphology and CNT forest internal structure (uniformity, alignment, etc.) were investigated. By controlling the FIB processing, a synergy between the top-down (shape of CNT metamaterial nanostructures) and bottom-up (structure of CNT forest) approaches is achieved.

## Experimental

A 30-nm-thick AlO_x_ support layer and a 0.9-nm-thick Fe catalyst layer were deposited using radio frequency (RF) magnetron sputtering on p-type (100) silicon substrates. The detailed procedure has been described previously [30]. The growth of CNTs was conducted using thermal chemical vapor deposition (CVD) on the deposited AlO_x_/Fe catalyst. The annealing process was performed at 730 °C in a hydrogen atmosphere with a gas flow rate of 65 sccm for 2.5 min (*p* = 29 Pa). After annealing, the CNT forest was grown in an acetylene (15 sccm) and hydrogen (15 sccm) atmosphere (*p* = 110 Pa), for a precisely controlled period of time.

Prior to CNT growth, the catalyst films were patterned into split ring resonator (SRR) shapes using a FEI QUANTA 3D 200i FIB system. The FIB processing parameters are summarized in Table 1.

**Table 1:** Parameters of FIB patterning

Parameter	Value
Beam current (pA)	30
Acceleration voltage (kV)	30
Ion beam source	Ga
FIB patterning depth (nm)	1, 1.5, 2, 4, 10
Secondary etching depth – whole area (nm)	0.2, 0.3, 0.4, 0.5
Ion beam diameter (nm)	17
Dwell time (s)	1.0 × 10^−6^
Overlap (%)	50

**Fig. 1:**
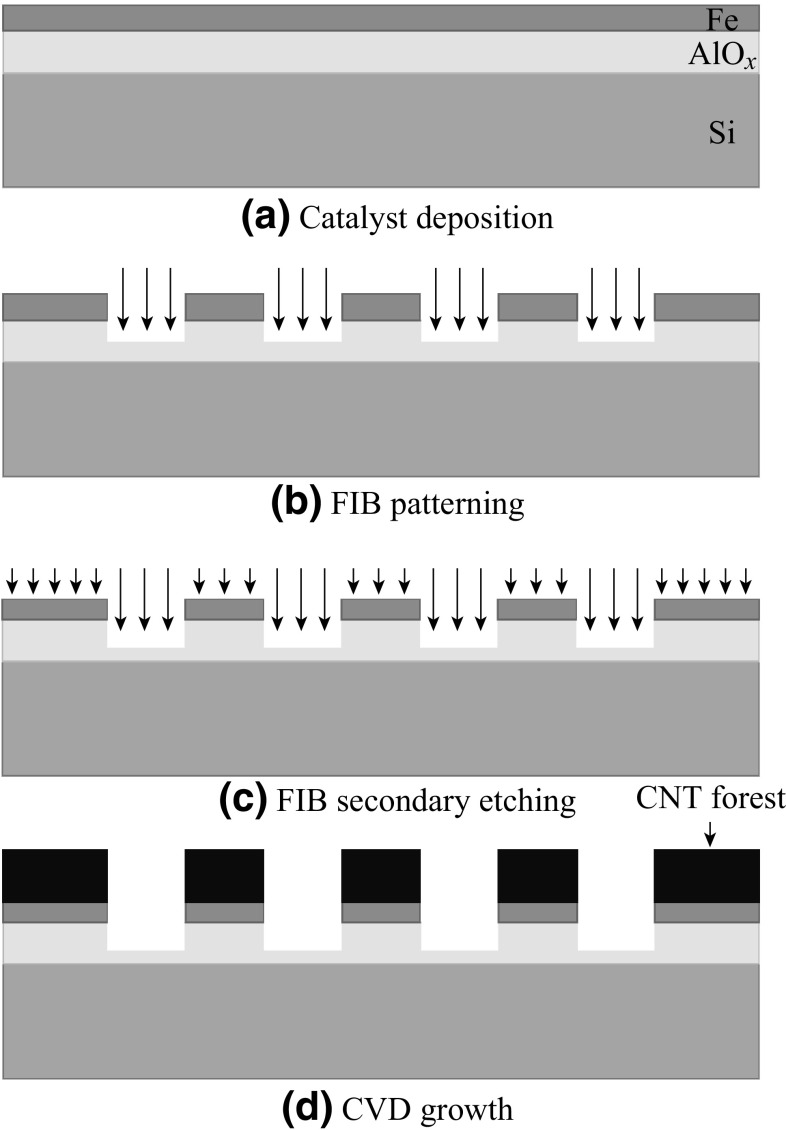
Schematic of process for CNT metamaterial fabrication: **a** Catalyst deposition, **b** FIB patterning, **c** FIB secondary etching, and **d** CVD growth

The experiments were conducted according to the following procedure: (1) deposition of AlO_x_/Fe catalyst on Si substrates by RF magnetron sputtering; (2) FIB patterning of metamaterial patterns on the catalyst, followed by (3) FIB secondary etching process, without breaking vacuum; (4) CVD growth of CNT on the prepared samples (Fig. 1).

**Fig. 2:**
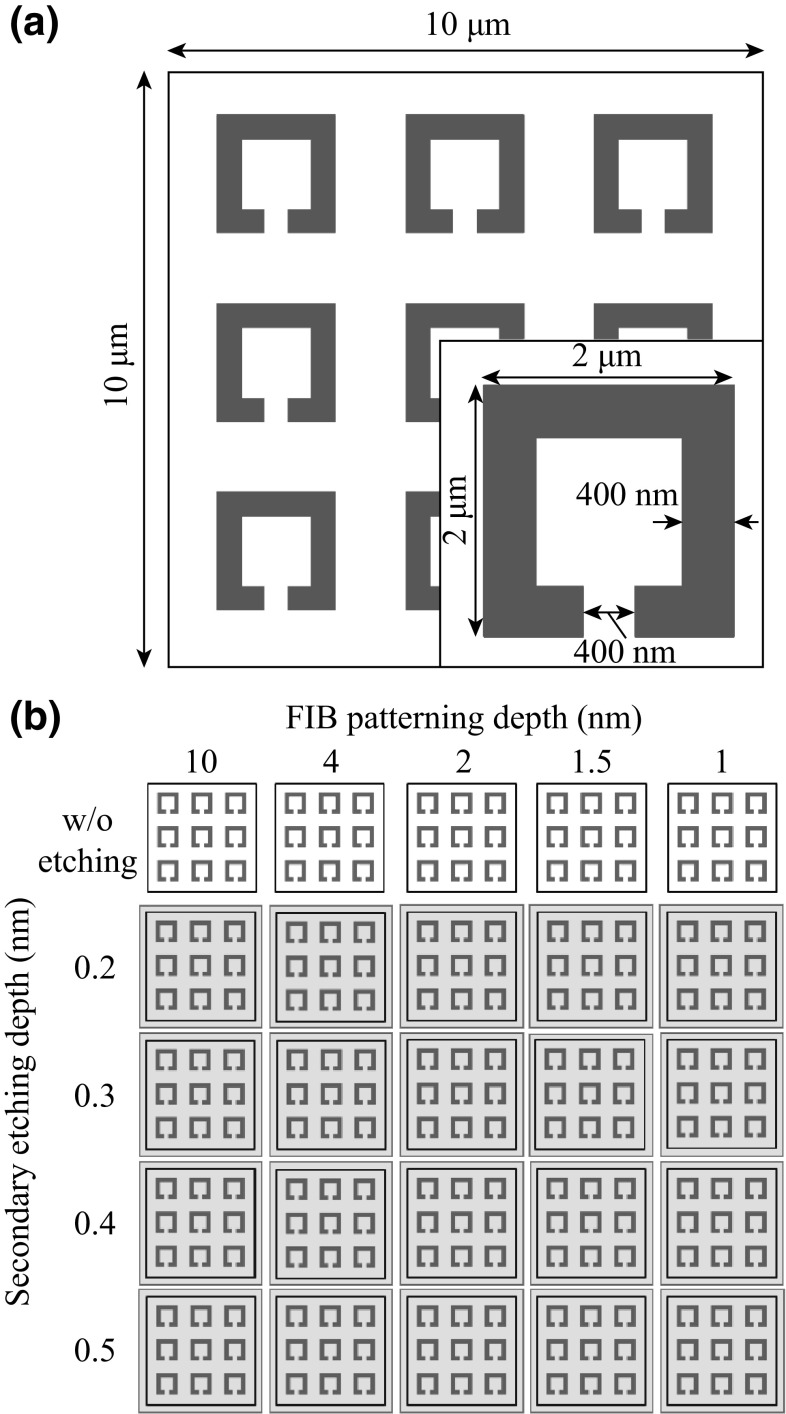
**a** Schematic of metamaterial patterns used for fabrication and **b** map of FIB milling with patterning and secondary etching depths

Figure 2a shows a design of the CNT metamaterial patterns used in FIB fabrication. The patterned area of 10 × 10 µm^2^ contained 9 SRR patterns of 2 × 2 µm^2^ size with a wall thickness of 400 nm. It should be noted that the FIB etching was selectively applied to the surface during the fabrication, to obtain non-etched SSR pattern areas (i.e., pristine deposited catalyst), while the secondary etching was applied to the entire surface, including SRR patterns. To study the effects of the patterning depth (1–10 nm) and the secondary etching depth (0.2–0.5 nm), a map containing 25 SRR pattern arrays was prepared (Fig. 2b).

The morphology of the catalyst surface was determined by using an atomic force microscope (AFM, SPI3800 N/SPA400, SII Nanotechnology Inc.), in the dynamic force microscopy (DFM) mode (tip curvature radius of 10 nm). The structure of patterns and morphology of CNT forests were characterized by using a field emission scanning electron microscope (FE-SEM, JEOL JSM-5310). The analysis of the type and quality of grown CNTs was performed by using a micro-Raman HORIBA JOBIN–YVON HR-800 spectrometer with laser excitation of 532.08 nm. A transmission electron microscope (TEM, JEOL JEM 2100 M) was used to observe the diameter of CNTs in the patterned arrays.

## Results and Discussion

**Fig. 3:**
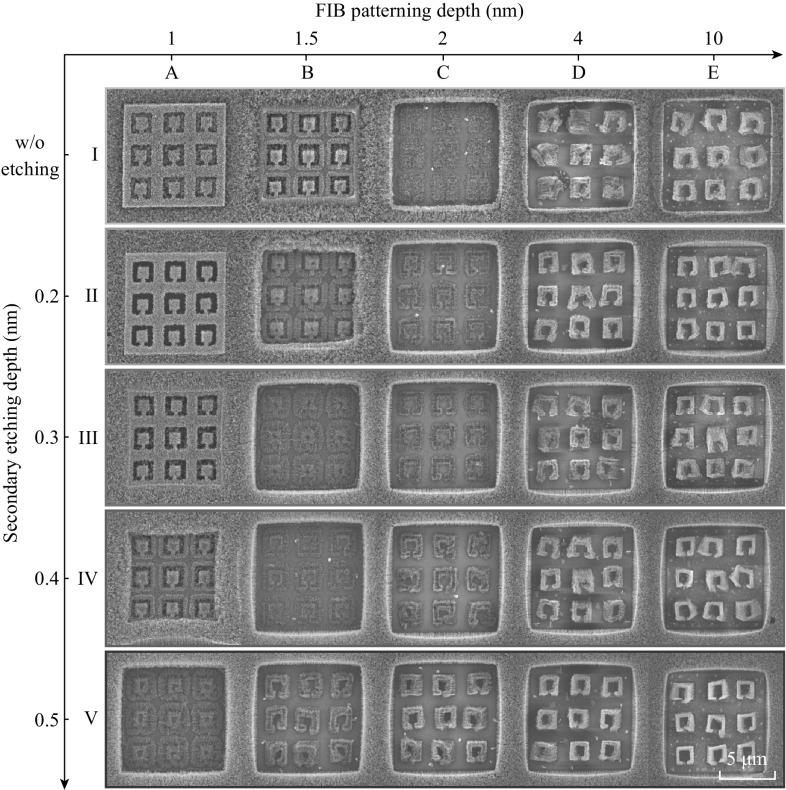
SEM images of patterned surface after CNT forest growth. *Arabic numerals* refer to depths in nanometers, while *letters* and *Roman numerals* were introduced for the purpose of analysis

The results of FIB patterning depth and FIB secondary etching depth are shown in Fig. 3. The patterning depth of 10 nm allowed the growth of CNT SRR arrays (Fig. 3 E(I–V)). The quality and density of CNTs in these patterns were improved by applying the secondary etching of 0.2–0.5 nm. A similar trend was observed for other patterning depths and was most noticeable for the patterning depth of 2 nm (Fig. 3 C(I–V)). Without the secondary etching, very low levels of alignment and randomly aligned CNTs were grown in the SRR patterns. After the introduction of the FIB secondary etching process, the pattern gradually appeared, as the more vertically oriented growth of CNTs was observed. Interestingly, in the area with a patterning depth of 1 nm, for secondary etching depths of 0.2 and 0.3 nm (Fig. 3 A(II–III)), inverted SRR arrays were observed. The high aligned growth of CNTs in SRR arrays for depths of 4 and 10 nm (Fig. 3 D, E) was successfully obtained, and a very small diameter of 2–3 nm was observed in a high-resolution SEM image (Fig. S1). It was established that the entire catalyst layer (0.9 nm thick) and part of the AlO_x_ support layer, including the diffused Fe catalyst, were completely removed in the case of patterning depths of 4 and 10 nm, leaving only non-etched SRR areas with the preserved catalyst and thereby allowing the growth of CNT forests. For a patterning depth of 2 nm (Fig. 3 C), short and randomly oriented CNTs were observed in SRR patterns by adding the secondary etching process (Fig. 3 C(II–V)). By reducing the patterning depth to 1 and 1.5 nm (Fig. 3 A(I), B(I)), without secondary etching, the growth of CNTs was observed in the etched area, while the growth of CNTs in the patterns was suppressed. It was found that in these cases, the patterning depths were not sufficient to completely remove the catalyst film from the surface by secondary etching, resulting in the increase in the growth height on the thinner catalyst [19], while the redeposited material suppressed the growth of CNTs in the designated SRR patterned areas. Applying secondary etching can remove the redeposited material from patterns, allowing the growth of randomly oriented CNTs. Thus, it was concluded that secondary etching affected the catalyst surface morphology and was the primary reason for changes in the CNT growth.

**Fig. 4:**
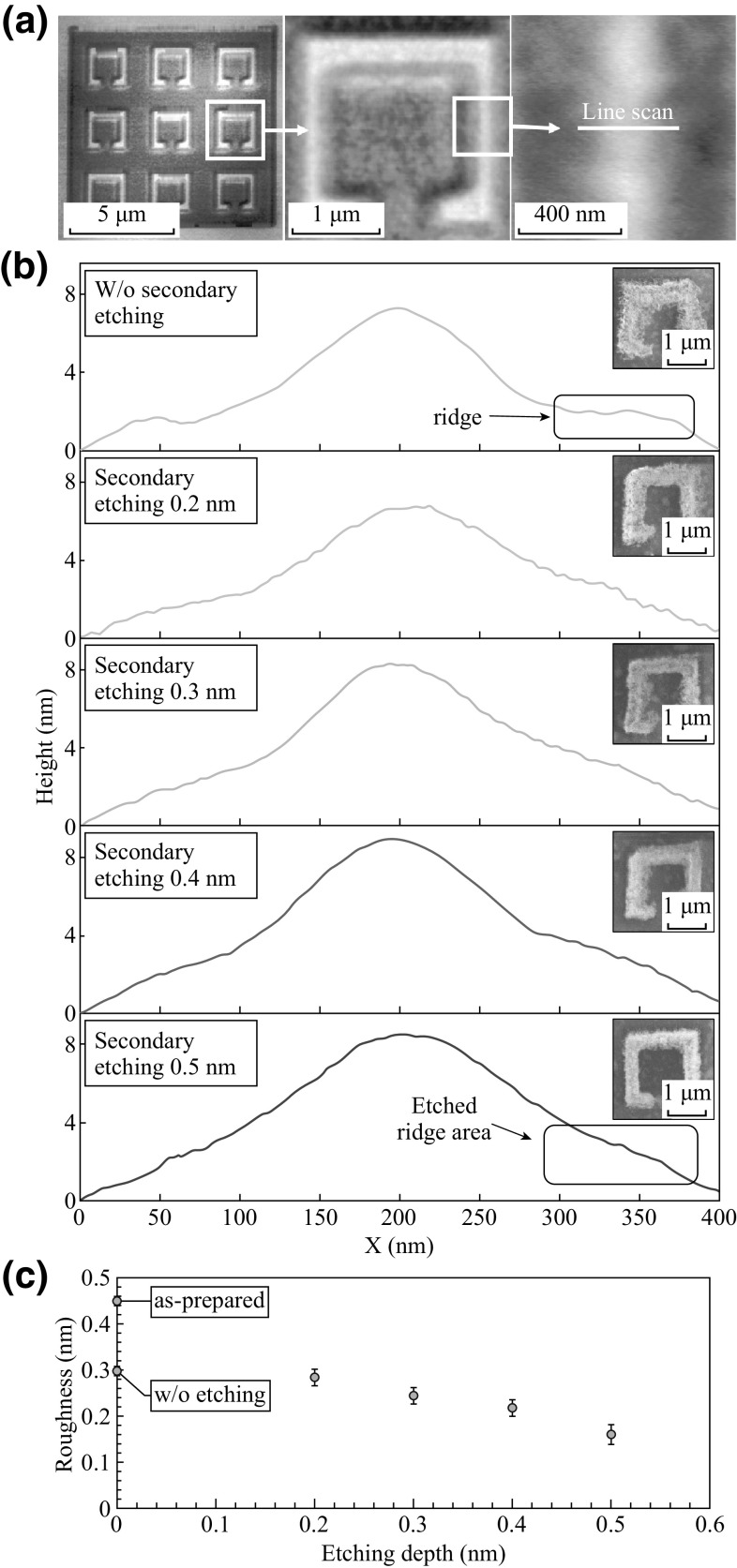
**a** AFM images of patterned catalyst, **b** line profiles of pattern with applied secondary etching (0–0.5 nm), and **c** roughness analysis of the catalyst surface

In order to investigate the secondary effect caused by the secondary etching, AFM analysis of the patterned area was conducted before CNT growth as shown in Fig. 4. The AFM image of fabricated patterns with a depth of 10 nm and secondary etching of 0.5 nm are shown in Fig. 4a. To examine the morphology of the patterned surface, line profiles of secondary etching (0–0.5 nm) were prepared (Fig. 4b).

As shown in Fig. 4b (w/o secondary etching), on both sides of the profile with around 50 and 350 nm, the formation of ridges was noticed as a result of resputtering during the FIB patterning. Similar ridges were observed in Refs. 15 and 29 after the FIB patterning process. By applying secondary etching to the surface, the top layer of material was removed, and the height of the ridges diminished to the point where they were no longer observable (for 0.4 and 0.5 nm etching depths). A more detailed analysis of the average roughness of the Fe catalyst surface, after FIB patterning and secondary etching, was conducted and is presented in Fig. 4c. For comparison, the average roughness of the as-prepared catalyst is also shown. The FIB patterning resulted in a decrease in the average roughness. It was assumed that the sputtered material redeposition on the patterns resulted in a smooth surface via uniform redeposition. Furthermore, by applying the FIB secondary etching process, the average roughness was further decreased, from *R*_a_ = 0.45 nm for an as-prepared catalyst surface to 0.15 nm for a secondary etching depth of 0.5 nm. Lower roughness supported the formation of uniform catalyst particles and resulted in the growth of high-density CNT forests.

**Fig. 5:**
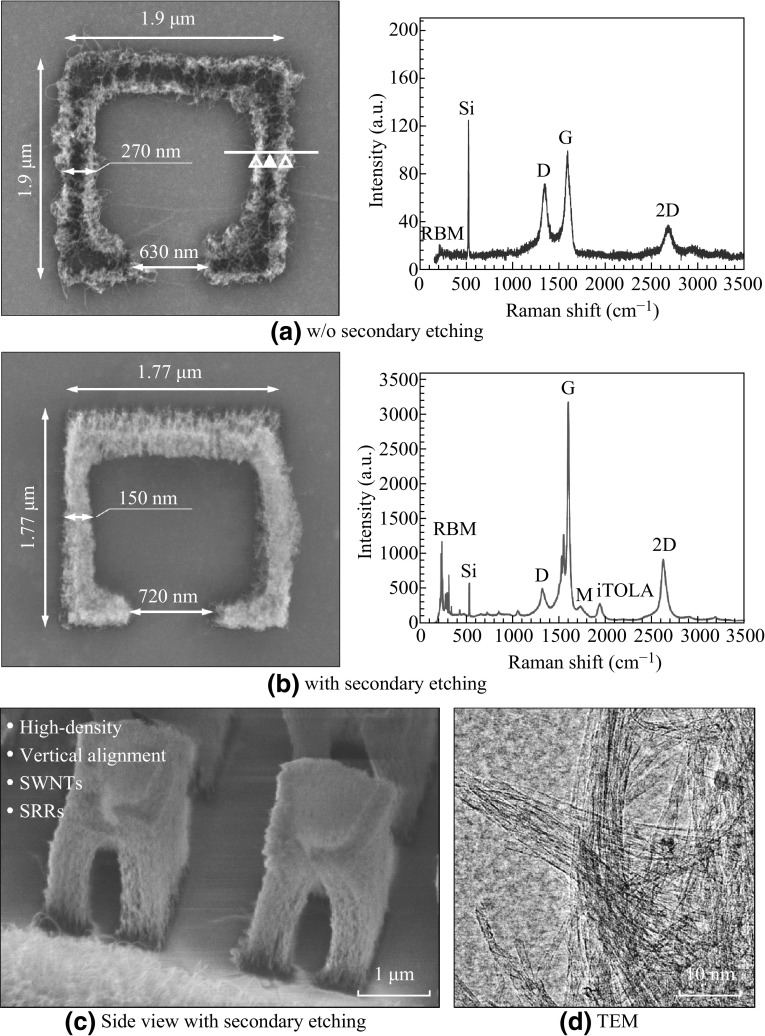
SEM images and Raman spectra of CNT patterns: **a** without secondary etching; **b** with secondary etching of 0.5 nm depth; **c** side view with secondary etching of 0.5 nm depth; **d** TEM image of a CNT array grown under the optimized FIB processing conditions shown in **c**

The effects of the removal of the redeposited material from SRR structures and the decrease in the average roughness after the secondary etching process were investigated (Fig. 5). In Fig. 5a, without secondary etching, based on the numerous areas in the CNT forest where there are no CNTs, the growth of the CNT forest on the patterns was found to be highly inhomogeneous with low CNT density and low alignment. The growth of CNTs was mostly observed on the edges of the fabricated patterns as shown in Fig. 5a. Furthermore, despite the fact that the as-prepared CNT forest was composed of SWNTs, the Raman spectra of the SRR pattern revealed a mixture of MWNTs and SWNTs. This was indicated by the G/D peak intensity ratio of 1.45 and very low signal of radial breathing mode (RBM), originating from the presence of SWNTs. In contrast, after the secondary etching for a 0.5 nm depth, the growth of CNTs was greatly improved as shown in Fig. 5b, c. Both the density and alignment, as well as the uniformity, improved. This suggests that the growth of CNTs occurs on the entire surface of patterns. Raman measurements of CNTs after the secondary etching showed the improvement of the CNT forest structure. For the relatively low height of the CNT metamaterial structures, the overall intensity was significantly improved with the same measurement parameters. Analysis of the Raman spectra showed a high intensity of RBM peaks, indicating a high number of SWNTs in the structure, while a significantly high G/D peak ratio of 10.47 indicated high crystallinity and a low number of defects in the graphene layers of the CNTs. This conclusion was also confirmed by a high-intensity 2D band. Finally, the presence of additional M, iTOLA, and G^−^ bands [31] was also observed in the Raman spectra, indicating the enhanced quality of graphene in the CNTs. The detection of Raman peaks of SWNTs was confirmed by TEM observation results, which revealed that the patterned CNT forest arrays contained SWNTs (Fig. 5d).

The reason for the differences between the cases, i.e., with and without secondary etching, was related to the annealing process at high temperatures. For the case without secondary etching, the surface diffusion of catalyst particles was limited by the layer of redeposited material at the ridges. The catalyst particles agglomerated in the area before the ridges, which became a barrier for further catalyst surface diffusion to the area outside the ridges, resulting in a higher area density of the particles, allowing the growth of CNTs on the edges.

On the other hand, the effect of secondary etching was determined by the amount of removed material and the decreased surface roughness. During the patterning of SRR arrays, the sputtered material was mainly redeposited on the edges of patterns, forming ridges (Fig. 4b), and, to a lesser degree, in the middle of the walls. Furthermore, it was assumed that the mass density of the redeposited film was lower than that of the catalyst film. So, the sputtering rate was higher, resulting in faster etching at the ridge area as shown in Fig. 4b. By applying secondary etching of 0.5 nm, the majority of resputtered material and the top layer of Fe catalyst film were removed. By additional ion irradiation during the secondary etching process, cleaning of the surface and a decrease in the thickness of the Fe catalyst is to be expected, and these result in a decrease in the roughness and a consequent improvement in the height, uniformity, and quality of CNTs. Finally, as a result of the etching, due to the thinner catalyst and significantly decreased roughness, the growth of thin SWNTs with a higher growth rate was achieved [19], which was also observed in Fig. 3 A(I–II).

In the present study, the 30 kV accelerating voltage of the Ga ion was the direct cause of sputtering and redeposition of the material in the patterned areas; however, the other possible effects should also be noted. High accelerating voltages may cause implantation and diffusion of the Ga ion caused by ion beam irradiation. During the FIB patterning process, the depth of patterning was sufficient to remove the catalyst from the designated area, resulting in no growth of CNTs. On the other hand, the FIB secondary etching treatment of the patterned catalyst was used to clean the surface of the redeposited material. During the process, most of the deposited catalyst remained, and despite a relatively low ion energy, the implantation of Ga ions was possible. However, due to the significant improvements in the CNT forest observed after the secondary etching, the possible influence of Ga ions was negligible and could be ignored.

Finally, as can be seen in Figs. 2 and 5, the size of the patterns was significantly decreased after FIB secondary etching. It was assumed that this shrinking effect was related to the FIB patterning process. During patterning, the ion beam was oriented perpendicularly to the surface and the surface material was sputtered. Typical patterns milled using FIB do not possess vertical walls and flat bottoms, but are V-shaped with a maximum depth in the middle [29, 32, 33]. Furthermore, the mouth width of these V-shaped patterns is usually much larger than the diameter of the ion beam. This effect can be explained by the high intensity of the ions outside the core region, which causes a relatively higher sputtering of the material on the sides of the walls, thus resulting in the creation of slopes. Therefore, with an increase in the etching depth, the duration of ion beam irradiation is also increased, and more material from the walls is sputtered. During the sputtering, a part of the surface with the pristine catalyst is also removed from the edges of the walls, causing overall shrinking of the patterns. This shrinking effect should be taken into consideration during the design of fine patterns of CNT forest metamaterials in the visible region.

## Conclusions

This work presents the combination of precise FIB patterning with additional secondary FIB etching in order to remove redeposited material from the surface and improve the growth of a CNT forest in the SRR array nanostructures. This method enables, for the first time, the fabrication of nanoscale metamaterial patterns and the catalytic growth of high-density CNT structures as small as about 150 nm on a predeposited catalyst film in designated areas. The patterning depth of 10 nm and the secondary etching depth of 0.5 nm allowed the growth of uniform, high-density, and highly aligned CNT forest metamaterials. The FIB secondary etching method decreased the average roughness of the catalyst surface, resulting in a significant improvement in the CNT forest quality. The top-down process of FIB patterning, allowed the precise fabrication of SRR structures; further, in the bottom-up approach for the fabrication of CNT forest metamaterials, the CNT forest growth could be controlled depending on the catalyst preparation. In the future, the effects of the redeposited material and of the catalyst film and the mutual interactions between them should be investigated to assist in the design of light wave-sized CNT metamaterials for optical, thermal, and optronic devices.

## Electronic supplementary material

Below is the link to the electronic supplementary material.

Supplementary material 1 (PDF 448 kb)
