# Supplementary material for: FIB Secondary Etching Method for Fabrication of Fine CNT Forest Metamaterials
Source: Nanomicro Lett. 2017 Apr 8;9(4):44. doi: 10.1007/s40820-017-0145-5 (PMC6199040; doi:10.1007/s40820-017-0145-5)
Supplement: Supplementary file 1 — Supplementary material 1 (PDF 448 kb) [file 40820_2017_145_MOESM1_ESM.pdf]

Supporting Information for

FIB Secondary Etching Method for Fabrication of Fine CNT Forest Metamaterials

Adam Pander[1,*], Akimitsu Hatta[1, 2], Hiroshi Furuta[1, 2]

[1]Department of Electronic and Photonic Systems Engineering, Kochi University of Technology, Tosayamada, Kami, Kochi 782-8502, Japan

[2]Center for Nanotechnology, Research Institute, Kochi University of Technology, Tosayamada, Kami, Kochi, 782-8502, Japan

*Corresponding author. E-mail: adampander1@gmail.com or 186001x@gs.kochi-tech.ac.jp

Tel: +81 (0)80 2983 4051

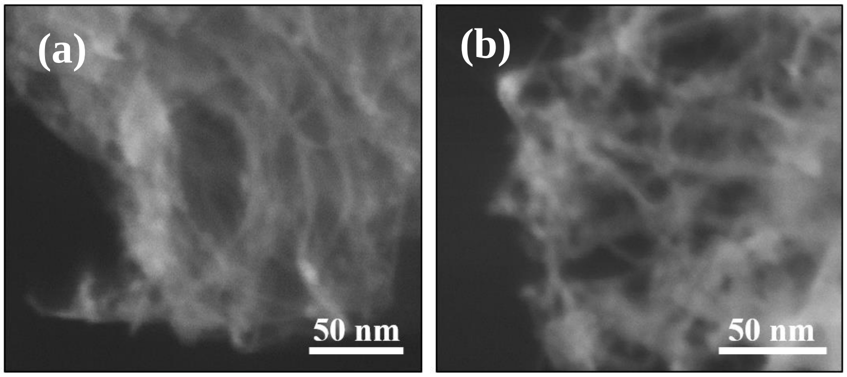

Fig. S1 High resolution SEM images of CNT arrays grown using patterning depth of 10 nm and FIB secondary etching depth of 0.5 nm. Magnification of **a** ×400 000, and **b** ×500 000
